# Enlarged Eye-Box Accommodation-Capable Augmented Reality with Hologram Replicas

**DOI:** 10.3390/s24123930

**Published:** 2024-06-17

**Authors:** Woonchan Moon, Joonku Hahn

**Affiliations:** School of Electronic and Electrical Engineering, Kyungpook National University, 80 Daehak-ro, Buk-gu, Daegu 41566, Republic of Korea; wcmoon@office.knu.ac.kr

**Keywords:** augmented reality (AR), waveguide, holographic display, computer generated hologram

## Abstract

Augmented reality (AR) technology has been widely applied across a variety of fields, with head-up displays (HUDs) being one of its prominent uses, offering immersive three-dimensional (3D) experiences and interaction with digital content and the real world. AR-HUDs face challenges such as limited field of view (FOV), small eye-box, bulky form factor, and absence of accommodation cue, often compromising trade-offs between these factors. Recently, optical waveguide based on pupil replication process has attracted increasing attention as an optical element for its compact form factor and exit-pupil expansion. Despite these advantages, current waveguide displays struggle to integrate visual information with real scenes because they do not produce accommodation-capable virtual content. In this paper, we introduce a lensless accommodation-capable holographic system based on a waveguide. Our system aims to expand the eye-box at the optimal viewing distance that provides the maximum FOV. We devised a formalized CGH algorithm based on bold assumption and two constraints and successfully performed numerical observation simulation. In optical experiments, accommodation-capable images with a maximum horizontal FOV of 7.0 degrees were successfully observed within an expanded eye-box of 9.18 mm at an optimal observation distance of 112 mm.

## 1. Introduction

Augmented reality (AR) technology has recently been widely utilized in a variety of fields, including industry, military, healthcare, education, and entertainment [1,2,3,4]. One of the most popular applications of this technology is head-up displays (HUDs), which offer a realistic and immersive experience that combines three-dimensional (3D) virtual contents with the real scenes [5,6,7]. However, AR-HUDs encounter several common challenges when satisfying the demands of human vision, including the field of view (FOV), eye-box, form factor, and accommodation cues. Moreover, these requirements entail tradeoffs with each other. Among these factors, the FOV affects immersion, realism, spatial recognition, user convenience, and task performance in AR systems. Accordingly, many studies have attempted to optimize FOV parameters to provide realistic and realistic experiences. For example, the magnifier-principle method employs basic refractive optics to magnify a micro-display to a virtual image [8]. Although this approach can be effective in providing an enlarged viewing angle, it is limited by not ensuring a compact system form factor because a relatively long optical path between the optical element and the display is required. Several alternative technologies have been explored to achieve a more compact form factor with a wide FOV, such as holographic optical elements [9], Pancharatnam–Berry phase lenses [10], and polarization-based folded lens, also known as pancake lenses [11]. However, these refractive optics-based approaches not only introduce chromatic aberrations but also cause significant optical aberrations and distortions in systems with a large FOV.

Recently, the waveguide approach has gained attention as an optical component that offers a compact form factor with a relatively large degree of design freedom. An additional key advantage of this approach is that pupil replication technology can provide a fairly large FOV without sacrificing eye-box. Given these advantages, the waveguide approach has recently emerged as the dominant technology for AR displays. The exit pupil replication process in a waveguide involves extracting a portion of light from the out-coupler and then directing it toward the observer. The residual light within the waveguide continues to propagate and interacts again with the out-coupler to extract some light. This iterative process creates numerous replicas of the input wavefield, effectively transforming the waveguide into a transparent periscope featuring a single entrance pupil and an expanded exit pupil. Therefore, expanding the exit pupil in the waveguide reduces the optical path length and provides a large FOV without sacrificing the eye-box. These waveguides can be designed using various types of optical coupling elements. For example, geometrical waveguides use a prism and transflective mirror array to redirect and extract light in the waveguide [12,13]. In diffractive waveguides, incident light enters the waveguide at an angle due to an inclined grating called the in-coupler. Light propagates within the waveguide and is expanded and extracted from the exit pupil through a second inclined grating, the out-coupler. Surface relief gratings, volume holographic gratings, polarization gratings, and meta-surfaces can be utilized as these in/out couplers [14,15,16].

Despite the advantages that waveguides offer as exit pupil expanders, their ability to provide practical and realistic visual results is limited. More precisely, conventional waveguide displays based on the pupil replication process only generate images with infinite depth. Because the pupil replication process in a waveguide creates numerous shifted copies of the input wavefield, the entrance pupil should be formed above the collimated field, regardless of the coupler type. When the display engine outputs the near field instead of the far field to generate a virtual image with finite depth, this results in complex fields that become scrambled and interfere with each other at the exit pupil of the waveguide. This interference results in visual artifacts, characterized by overlapping of the same image at different depths of focus. Since virtual images are rendered at optical infinity regardless of their perceived spatial location, the vergence-accommodation conflict (VAC) problem arises because of the depth mismatch between virtual and real objects [17].

Although various approaches have been attempted to solve the VAC problem that arises in these waveguide displays, they still face significant limitations. For example, in some studies, 3D voxels were expressed by masking sub-regions of elemental images corresponding to overlapping voxels in order to optimize the emission angle of integrated voxels. However, this method not only degrades the resolution of the voxels due to image masking, but also limits the depth range of voxels that can be expressed [18]. Other research has explored a pair of tunable lenses with complementary focuses to project a tomographic virtual 3D image, but this has issues such as optical aberrations caused by the lenses and not being able to generate multi-depth images [19].

In this respect, holography technology, which reconstructs 3D images by precisely manipulating the wavefront, is considered the most promising. Compared to methods such as light field display [20,21], multi-depth display [22,23], and Maxwellian-view display [24,25], holography provides essential 3D visual cues such as accommodation-vergence matching [26,27]. Meanwhile, current holographic displays suffer from several challenges caused by the limited capabilities of spatial light modulators (SLMs), which are essential devices for modulating the amplitude as well as phase of the wavefield. Typically, commercial SLMs do not accomplish fine pixel pitch or sufficient pixel count, resulting in a limited FOV and small eye-box issues in holographic displays. One useful way to improve the quality of reconstructed holographic images is to spatially concatenate multiple SLMs in a holographic system, which achieves a large FOV without sacrificing eye-box size. However, this increases the cost and form factor, while also requiring precise alignment and calibration of individual SLMs to generate seamless images without artifacts between individual SLMs [28,29]. In addition, a neural network parameterized wave propagation model for waveguide holographic near-eye displays is developed, resulting in high-quality holographic images with artifacts removed. However, this study is limited to near-eye displays only and unfortunately suffers from small eye-box [30].

In our previous work, we proposed a lensless holographic waveguide system consisting of a waveguide, a phase-only SLM, and a laser light source. In this waveguide-based holographic display, we defined virtual SLM replicas generated by the pupil replication process and utilized virtual SLM replicas to successfully generate accommodation-capable holographic images with maximum FOV. This technique solved the VAC problem by generating accommodation-capable holographic images over the full depth range [31]. Despite this being a promising technology to extend the FOV for accommodation-capable holographic images at a fixed observation distance in a waveguide-based holographic system, it faces the drawback of a reduced eye-box. This limitation originated from the inherent trade-off between the FOV and eye-box size imposed on an SLM. So, an enlarged eye-box while extending the FOV of the accommodation-capable image in waveguide-based holographic system is very challenging. There are two main methods for alleviating the limited eye-box size problem: temporal multiplexing and spatial multiplexing methods. Temporal multiplexing methods use fast-moving optical elements, such as LED arrays or dynamic mirrors, to dynamically alter the spatial position of the eye-box [32,33]. Spatial multiplexing methods use hologram optical element to expand the eye-box by generating multiple shifted pupils simultaneously [34]. The limitation of the multiplexed eye-box is manipulating the position or replication of the exit pupil without changing its intrinsic size, creating separate view-points within the dilated effective eye-box.

Accordingly, in this paper, we introduce a new method to expand the eye-box to overcome the sacrifice of eye-box at optimal observation distance and the maximum FOV in the lensless accommodation-capable holographic waveguide system. Other studies expanding the eye-box have relied on various techniques to enlarge the eye-box, including adjusting the viewpoint using a moving dynamic mirror and generating multiple viewpoints using holographic optical elements. However, these methods require mechanical movement or additional optical components. In comparison, our research offers a distinct advantage because the pupil replication process, which is driven by the waveguide used in the previous system, generates additional virtual SLM replicas to define multiple eye reference points. With the aid of eye tracking and software control that updates the computer-generated hologram (CGH) without mechanical adjustments, multiple eye reference points contribute to eye box expansion, producing an accommodation-capable image with the maximum FOV. Numerical simulations and optical experiments validate the success of the proposed system in reconstructing holographic images with the maximum FOV across the entirety of the expanded eye-box.

## 2. System Configuration and Method

### 2.1. System Configuration

The VAC problem was solved in our previous work by defining each copy of the modulated wavefield formed over the exit pupil of the waveguide as a virtual SLM and devising an algorithm to compensate for the focal spread of the replication fields. This method successfully generated both finite and infinite depth images in a waveguide-based AR system. The FOV plays an important role in determining the visible extent of the virtual environment and significantly influences immersion. In addition, the size of the eye-box is pivotal for depicting the user’s head and eye movement range while maintaining virtual content visibility, which affects usability and adaptability in various real-world environments. Unfortunately, although the developed method was successful in producing accommodation-capable 3D images with an extended FOV, this achievement was accompanied by a significant compromise of reducing the eye-box size due to the finite pixel resolution of the SLM. A narrow eye-box can limit the range of movement of a user’s head and eyes while reducing clear visibility of virtual content, adversely affecting both user comfort and the overall immersive experience. Consequently, the core goal of the current study is to not only construct a sufficiently wide FOV of the reconstructed image but also expand the sacrificed eye-box by forming multiple eye reference points.

**Figure 1:**
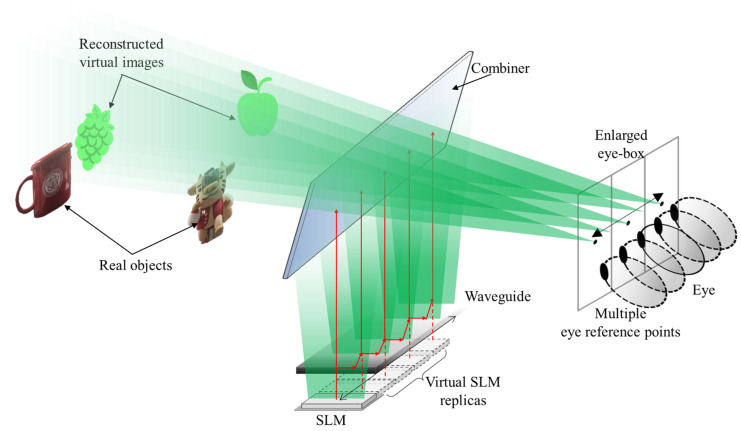
Configuration of proposed waveguide-based AR system with expanded eye-box.

Figure 1 displays the proposed waveguide-based AR system with an expanded eye- box, which consists of a laser light source, a single SLM, a waveguide used as a pupil expander, and a combiner. The collimated laser light source is spatially modulated by a computer-generated hologram loaded into the SLM and then projected onto the waveguide. The optical experiment may include a 4*f* optical system to eliminate DC noise and high-order diffraction terms resulting from the limited fill factor and individual pixel structure characteristics of the commercial liquid crystal panel used as an SLM. From the pupil replication process, a portion of the modulated field is extracted over the exit pupil and the remaining field continues to propagate within the waveguide. This process is repeated several times to create replicated virtual SLMs. The extended field extracted from the waveguide is then transferred to the observer as a fusion of the real scene and virtual images, which is achieved by a transflective mirror-type combiner with no optical power. Distinct from conventional waveguide displays that only produce virtual images with infinite depth, our system integrates accommodation-capable virtual images encompassing the full depth range with real scenes, offering users a more realistic and interactive experience. In addition, an algorithm is devised to establish virtual SLM replicas by employing a pupil duplication process. This method aims to create multiple eye reference points, effectively broadening the eye-box and facilitating extended an FOV of virtual images.

### 2.2. Optimal Observation Distance

The FOV is defined as an angular or distance range over which an object extends within an observer’s perspective. In a lensless holographic display system, the exit pupil is typically located at the SLM. Since the field is directly modulated by an SLM loaded with a Fresnel or Fourier hologram, the size of the exit pupil is limited to the size of the SLM itself. As a result, the maximum diffraction angle of the SLM determines both the maximum size of the holographic image and the system FOV. The size and position of the system’s exit pupil are closely related to the eye reference point, which represents the specific location in space where the viewer’s eye is expected to be located for optimal recognition of the holographic image. Essentially, the eye reference point is the location where light emerging from the exit pupil commonly converges. When the observer is aligned with the eye reference point, the holographic image can be observed with a maximum system FOV. However, when the observer’s position deviates from this eye reference point, the visible FOV is reduced. Therefore, the optimal observation distance in a holographic display system is established as the distance between the SLM and the eye reference point, where the viewer can observe a holographic image that offers the maximum FOV. An alternative approach to solving this problem involves using multiple SLMs arranged in a tile configuration to improve the freedom of the optimal observation distance. However, this requires complex optics and calibrations between individual SLMs to generate seamless images without noticeable artifacts. Accordingly, these systems invariably result in the development of bulky and intricate optical setups, rendering them incompatible with achieving a compact form factor.

**Figure 2:**
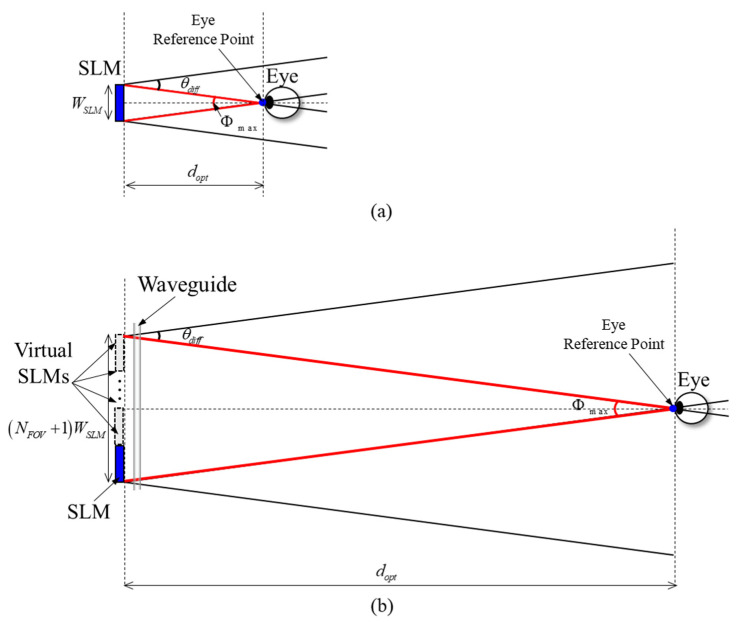
Definition of the optimal observation distance providing maximum FOV in a lensless holographic system (**a**) without waveguide and (**b**) with waveguide.

In a typical holographic system, the optimal observation distance is determined by the focal length of the eye-piece lens placed in front of the SLM. In contrast, in a lensless holographic system, the optimal observation distance is determined by the characteristics of the SLM. In particular, the farther the observation distance is from the optimal observation distance, the smaller the FOV becomes. Therefore, to formalize the expansion of FOV in our previous paper, FOV is a function of observation distance, and in this section, FOV is specified as the maximum FOV to derive the optimal observation distance. Alternatively, virtual SLMs generated through the pupil replication process enlarge the system’s exit pupil, positioning the eye reference point at a greater distance from the physical location of the SLM. Figure 2 shows the definition of the optimal observation distance providing maximum FOV in a lensless holographic system. Here, we focus on the one-dimensional case, as virtual SLMs are generated in the horizontal direction. Figure 2a depicts the principle of encompassing the maximum FOV at an optimal observation distance through the complete utilization of the bandwidth capacity of a single SLM. The SLM modulates the wavefront of an incident plane wave within the maximum diffraction angle range defined by $\theta_{\mathrm{diff}}=2\sin^{-1}\left(\lambda /2p\right)$. Here, λ is the wavelength of the laser light source, and p is the pixel pitch of the SLM. When the viewer moves away from the designated observation location, the bandwidth for object reconstruction diminishes, causing part of the image to become invisible and a reduction in the FOV. This issue originates from the limited diffraction angle and the size of the SLM. Figure 2b presents a waveguide-based holographic system utilizing a waveguide to create an array of multiple virtual SLMs. This planar array of virtual SLMs enhances the freedom of the optimal observation distance by enlarging the aggregate size of the SLM while maintaining the same diffraction angle. The optimal observation distance $d_{opt}$, at which a viewer can observe the entire holographic image with the maximum FOV, can be calculated from the following:

(1) $$d_{opt}=\frac{\left(N_{FOV}+1\right)W_{SLM}}{2\tan\left(\Phi_{max}/2\right)},$$

where $W_{SLM}$ is the width of SLM, $N_{FOV}$ denotes the number of virtual SLMs required to observe the entire holographic image at the optimal observation distance, and $\Phi_{max}$ represents the maximum FOV, and its value is equal to the diffraction angle of the SLM. Hence, the flexibility to adjust the optimal observation distance is determined by the number of virtual SLMs produced by the waveguide. The proposed waveguide-based holographic system offers a considerable degree of freedom in terms of the optimal observation distance. In addition, it generates accommodation-capable holographic images, effectively addressing the VAC problem prevalent in conventional systems. Section 3 presents a detailed description of the algorithm for calculating the CGH when implementing the proposed method.

### 2.3. Eye-Box Expansion

In a lensless holographic system, the eye-box is the space where the entire FOV of an image can be viewed without vignetting, and it is determined by a combination of the exit pupil size and the optical observation distance. This space represents the positional tolerance of the user’s eyes. Within the eye-box, viewers can move their heads and change their gaze angles while the entire reconstructed content remains clear and complete. Accordingly, ensuring a sufficiently large eye-box that exceeds the natural range of eye movement is important for user comfort and an immersive experience.

**Figure 3:**
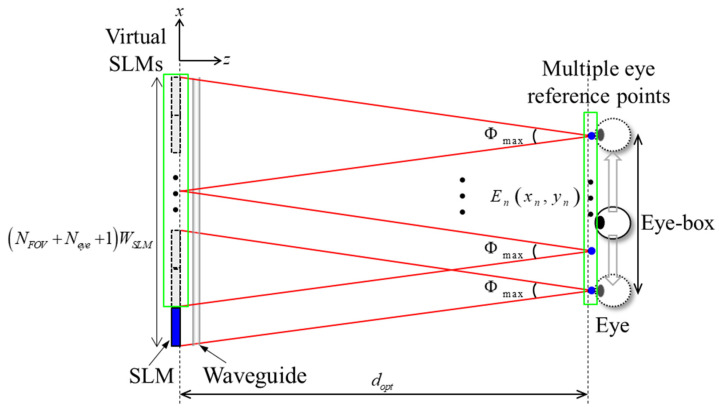
Principle of generating multiple eye reference points through the pupil replication process for eye-box expansion.

Our approach for expanding the eye-box in a lensless accommodation-capable holographic waveguide system has the advantage of generating multiple eye reference points by directly expanding the exit pupil of the system through a pupil replication process. In the multiplexing method, when the eye is located between neighboring viewpoints, part of the image is cut off or repeated images overlap, causing visual distortion. In contrast, our eye-box enlargement method supports effective eye-box expansion by providing continuous viewpoints within the entire range between the first eye reference point and last eye reference point. Figure 3 shows the principle of generating multiple eye reference points through the pupil replication process for eye-box expansion in a lensless holographic waveguide system. The total number N of virtual SLMs created through the replication process by the waveguide is given in the following:

(2) $$N=N_{FOV}+N_{eye}.$$

Here, $N_{eye}$ denotes the number of additional virtual SLMs tasked with creating multiple eye reference points. Since the eye reference point is defined based on each virtual SLM, the generated eye reference point may not be continuous, which is compensated for through the dynamic adjustment of the CGH using eye-tracking technology. By continuously updating the CGH as the eye moves, the system forms a series of viewpoints within the eye-box. The number of eye reference points generated in our system is $\left(N_{eye}+1\right)$, and the position of the nth eye reference point is expressed as follows:

(3) $$E_{n}\left(x_{n},z_{n}\right)=E_{n}\left(\frac{\left(2n-1+N_{FOV}\right)W_{SLM}}{2},d_{opt}\right).$$

The eye reference point is valid when $1\leq n\leq N_{eye}$, with n being an integer. Therefore, the eye-box size expanded through the pupil replication processes is calculated as follows:

(4) $$W_{eye}=\left|E_{1}E_{Neye}\right|=N_{eye}W_{SLM}.$$

Here, $\left|\cdot \right|$ denotes the Euclidean distance, which is a measure of the straight line distance between two points.

## 3. Eye-box Expansion Algorithm in Holographic Waveguide System

### 3.1. Optical Equivalent Modeling

In conventional waveguide-based displays, the generation of near-field images by the pupil replication process results in ghost artifacts where the same image overlaps at different depths of focus. This limitation originates from the intrinsic design and optical properties of the waveguide, which are optimized for projecting images at an infinite depth. Herein, we consider an approach to model the optical equivalent of a waveguide as a virtual SLM array to interpret the complex phenomena occurring within the waveguide.

**Figure 4:**
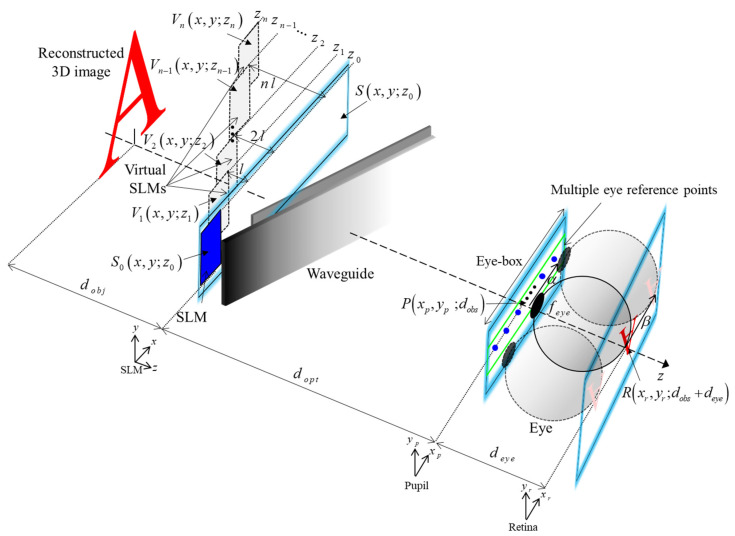
Optical equivalent modeling for expanding eye-box in a lensless holographic waveguide system.

Figure 4 depicts the optical equivalent modeling for expanding the eye-box in a lensless holographic waveguide system. This modeling strategy involves optically simplifying a single real SLM and waveguide into multiple SLMs, removing the physical complexity of the waveguide and focusing on the interaction between the SLM and the eye. This allows a direct transformation between the SLM and the eye, removing all intermediate space except the SLM and the eye in the forward and inverse cascaded Fresnel transforms (CdFr and ICdFr, respectively). The proposed model requires the bold assumption that N SLM replicas are effectively approximated by a single ultra-resolution SLM, which imposes two constraints. Here, the single ultra-resolution SLM has the same pixel pitch but has N times more pixels than the real SLM. One of the previously mentioned constraints is that the optical path from the eye is accumulated as l every time the virtual SLM is replicated. Another is that the calculated overall wavefield obtained in the SLM plane should be modulated only by the real SLM. Reflecting on the practical conditions caused by these constraints is essential for maintaining the bold assumption that underlies the model. Based on this optical equivalent modeling, the CGH that expands the eye-box at the optimal observation distance in the waveguide holographic system is calculated (see Appendix A).

### 3.2. Numerical Observation Simulation

From the mathematical relationship between the wavefield in the SLM plane and the wavefield in the retinal plane of the eye, the CGH is calculated by ICdFr, while the numerical observation simulation is performed by CdFr. In order to perform accurate observation simulation in the proposed system, it is necessary to obtain complex wavefield implemented in the SLM plane. The CGH pattern calculated in Equation (A6) and uploaded to the real SLM is formed repeatedly in the horizontal direction by the pupil replication process of the waveguide. Repetitive CGH patterns are created at the location where the virtual SLMs are formed, and the duplicated CGH patterns are propagated from the virtual SLM plane to the SLM plane. The entire wave field in the SLM plane, $S\prime \left(x,y;z_{0}\right)$, is calculated as follows:

(5) $$S\prime \left(x,y;z_{0}\right)=\sum_{n=0}^{N}ASM\left\{G_{SLM}\left(x+\frac{NW_{SLM}}{2}-nW_{SLM},y;z_{n}\right),-z_{n}\right\}.$$

The numerical observation result is derived by CdFr of the wavefield $S\prime \left(x,y;z_{0}\right)$ in the SLM plane, and the wavefield $R\prime \left(x_{r},y_{r};d_{obs}+d_{eye}\right)$ obtained in the retina plane considering the pupil size and pupil position of the eye is as follows:

(6) $$R^{\prime }\left(x_{r},y_{r};d_{opt}+d_{eye}\right)=CdFr\left\{S^{\prime }\left(x,y;z_{0}\right);d_{opt},d_{eye},f_{eye},\rho ,\alpha \right\},$$

where ρ is the radius of the pupil.

**Figure 5:**
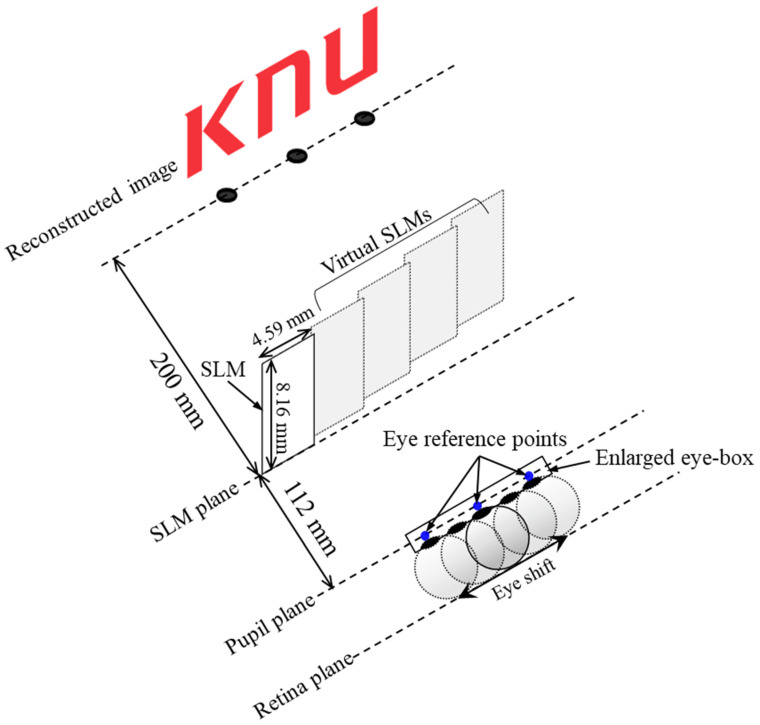
Numerical observation simulation that reconstructs an image of finite depth within an enlarged eye-box.

Figure 5 displays a numerical observation simulation to reconstruct an image of finite depth within an extended eye-box by applying the proposed algorithm. The CGH generated by Equation (A6) is numerically observed at the retina plane by Equations (5) and (6). In this simulation, the waveguide is optically replaced by four virtual SLM replicas, which create three eye reference points at the pupil plane. At an optimal observation distance of 112 mm set by Equation (1), this configuration allows an image to be reconstructed at a finite depth with maximum FOV within the extended eye-box. The reconstructed image with a depth of 200 mm is the Kyungpook National University logo composed of the letters “K”, “N”, and “U”. The detailed parameters used in the simulation are listed in Table 1.

**Figure 6:**
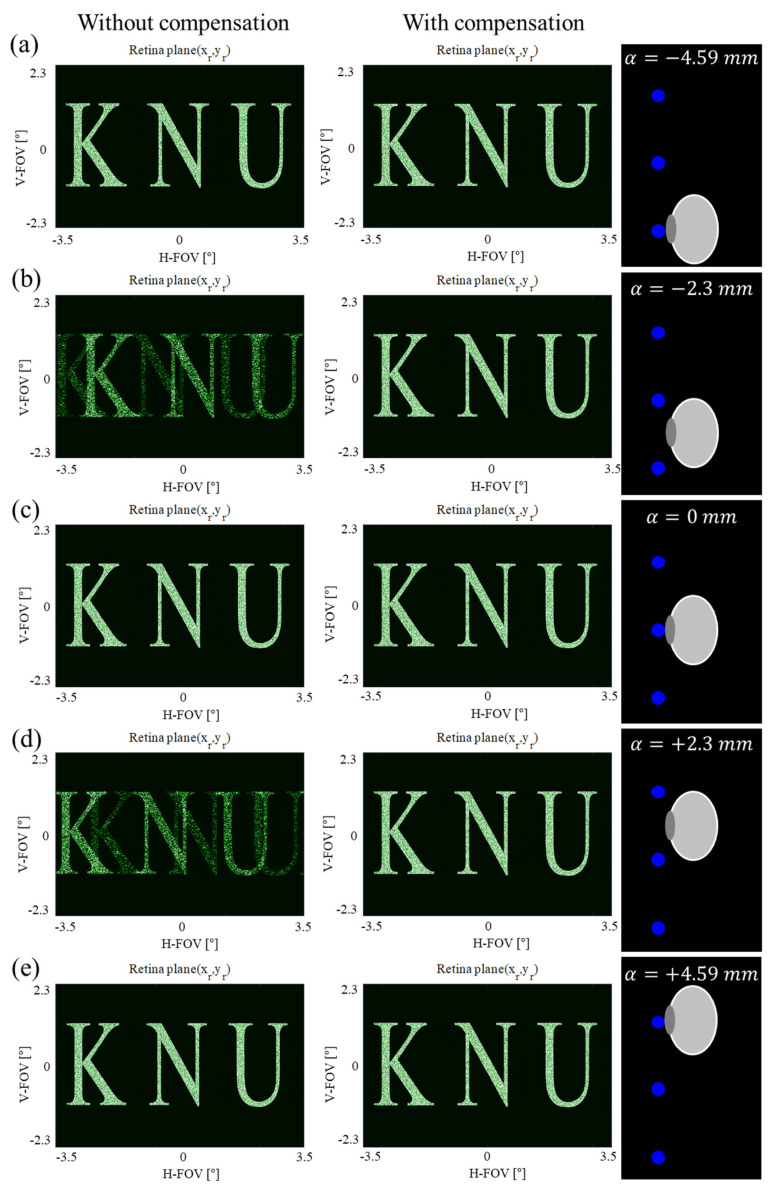
Numerical observation simulation results obtained without compensation (first column) and with compensation using eye tracking technology (second column) for shifted eye positions within the extended eye-box: (**a**) α=−4.59 mm, (**b**) α=−2.3 mm, (**c**) α=0 mm, (**d**) α=+2.3 mm, and (**e**) α=+4.59 mm.

The CGH calculated by the proposed algorithm in a lensless holographic waveguide system successfully implemented an expanded eye-box in numerical observation simulation. The first column of Figure 6 depicts the numerical observation results with CGH calculated without considering the moved eye position within the expanded eye-box. This case is the CGH generation method described in our previous paper [31], which assumes that the eye position is fixed due to the reduced eye-box. On the other hand, the second column of Figure 6 depicts numerical observation results of CGH compensated by the algorithm described in Section 3.1 for shifted eye positions within the expanded eye-box. This case uses eye-tracking technology to obtain information of the position of the moving eye and calculates different CGHs for the position of the shifted eye. When the eye is positioned at the eye reference point, almost no artifact is observed in the reconstructed image with and without compensation (Figure 6a,c,e). Because the optical path length difference between virtual SLMs is relatively very small, this is not expected to have a significant impact on the simulation result. There are gaps between neighboring eye reference points of size $E_{n}-E_{n-1}=W_{SLM}$, as depicted in Section 2.3. In particular, when the eye is located between adjacent eye reference points, the compensation effect of the proposed algorithm is clearly visible. Figure 6b shows the numerical observation results when the eye moves −2.3 mm from the fixed position. In the first column of Figure 6b, significant artifacts are observed, including unnecessary overlapping images. On the other hand, as shown in the second column, the correct image is observed by CGH compensated by applying eye tracking technology. As shown in Figure 6d, the same result is obtained when the eye position is moved to the opposite direction.

## 4. Optical Experiment Results

**Figure 7:**
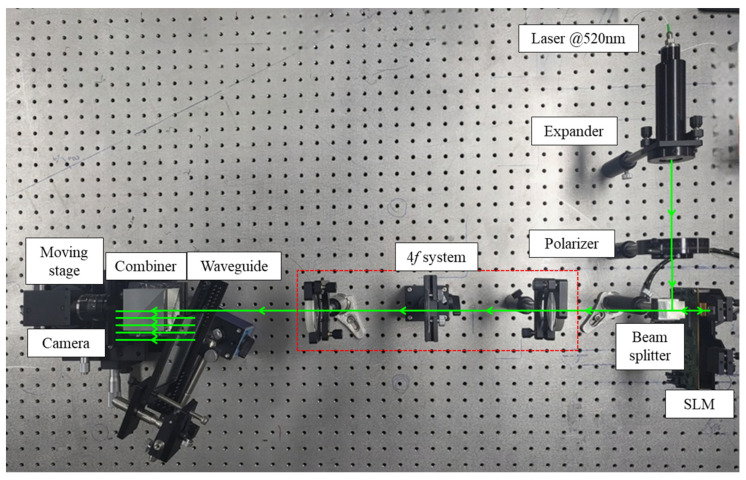
Experimental setup.

To verify the eye-box expansion method in the lensless holographic waveguide system on an optical table, an optical experiment was conducted as depicted in Figure 7. The optical experiment consisted of a laser light source, a single SLM, and a waveguide. The expander was used to expand the beam size of the fiber-coupled laser with wavelength of 520 nm. A liquid crystal on silicon (LCoS) device, model HX-7322, featuring a resolution of 1080 × 1920 pixels, was employed for phase modulation. The camera was located at the optimal observation distance and was equipped with a moving stage to mimic eye movements precisely. Although the experimental setup did not include an external eye tracking system, the eye pupil position is moved by an equipped moving stage and the CGH adjusted to display the holographic image. For reliable experiments, a camera similar to the optical characteristics of the eye was selected. Based on the camera’s f-number and the prime lens’ focal length, the aperture size of the camera was calculated to be 3.9 mm. The practical aperture ratio of commercial LCoS devices inevitably introduces unmodulated DC noise at the center of the reconstructed image. In optical experiments, this noise is a major factor causing errors with numerical simulation results, so it is removed optically in the 4*f* system.

**Figure 8:**
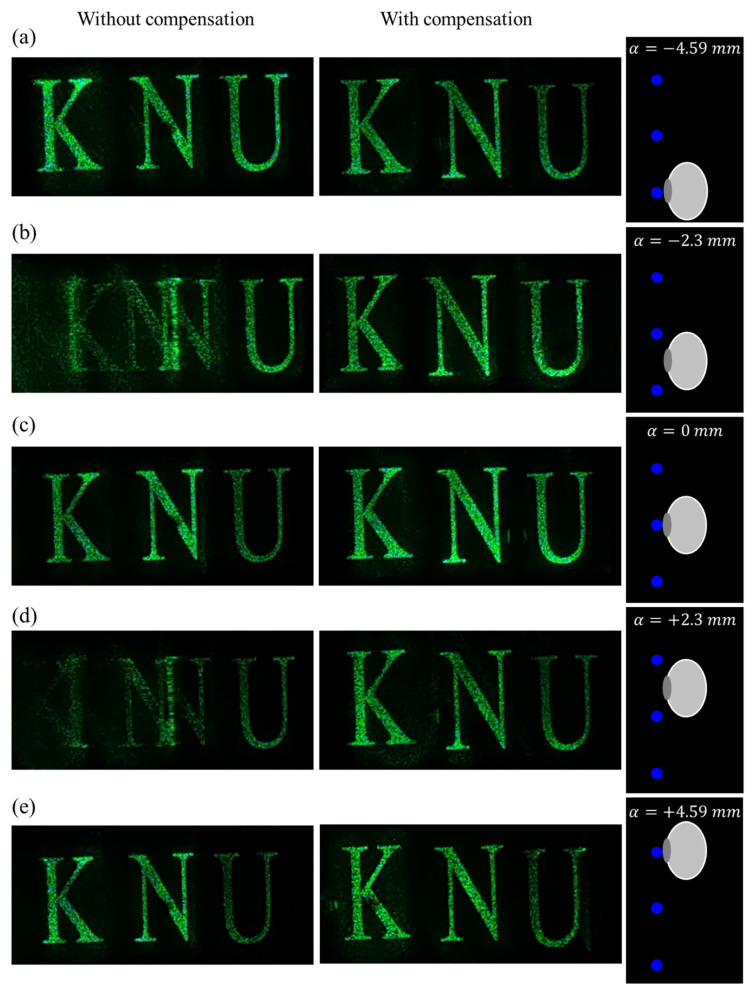
Optical experiment results captured without compensation (first column) and with compensation using eye tracking technology (second column) for shifted eye positions within the extended eye-box: (**a**) α=−4.59 mm, (**b**) α=−2.3 mm, (**c**) α=0 mm, (**d**) α=+2.3 mm, and (**e**) α=+4.59 mm.

The optical experiment results show that the eye-box was successfully expanded under the same conditions as the numerical simulation performed with in the previous section. In the experiment, eye movement within an extended eye-box was performed using a moving stage equipped with a camera. Based on the shifted eye position information, the compensated CGH was recalculated and loaded on the SLM. Figure 8 displays the optical experiment results captured for shifted eye positions within extended eye-box. The focal length of the camera is adjusted to focus the reconstructed image located 200 mm away from the SLM. The first column of Figure 8 displays images captured at shifted eye positions without compensation for the CGH calculated from fixed central eye position within the expanded eye-box. On the other hand, the second column of Figure 8 shows the images compensated by CGH recalculated from the shifted eye position using eye tracking technology. If compensation for the moved eye position is not applied as shown in first column of Figure 8, the eye movement results in a magnification error in the image, causing the image to enlarge or shrink depending on the direction of eye movement. Furthermore, significant ghosting artifacts occur when the eye is located between adjacent eye reference points. The CGH is compensated by the proposed algorithm in order to eliminate these artifacts, as shown in second column of Figure 8, ensuring that consistent and accurate images are observed within the expanded eye-box. These optical experimental results are almost consistent with the numerical simulation results and support the performance of the proposed algorithm. Ideally, observation results compensated by the algorithm within an expanded eye-box should perfectly match those observed at a fixed central eye position without compensation. However, a fiber-coupled laser with a high numerical aperture used as a light source in optical experiment does not provide an ideal collimated beam shape and exhibits an uneven brightness distribution throughout the beam profile. The incident beam with such a deviation causes differences in brightness of the captured image as the eye position moves. We plan to solve artifacts by improving the brightness uniformity of the entire beam profile of the collimated light source.

**Figure 9:**
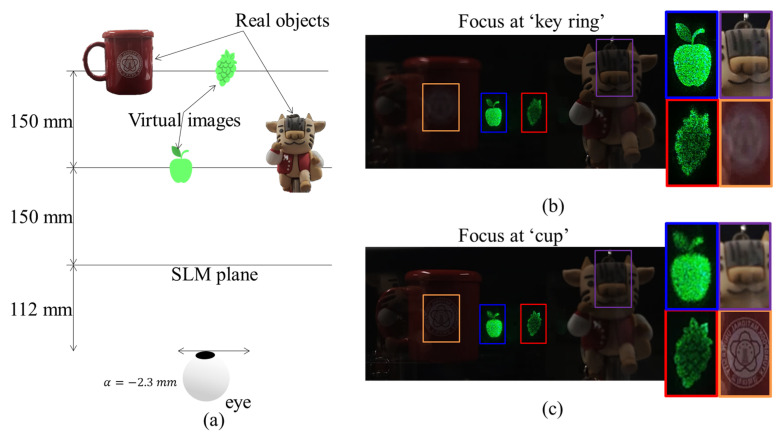
Experimental results of AR application: (**a**) original depth of real objects and virtual images, and captured pictures when the camera focuses at (**b**) a near object, i.e., a keyring, and (**c**) far object, i.e., a cup for fixed eye position. Note that the red and blue rectangular lines respectively indicate virtual contents at different depths, and likewise the orange and purple rectangular lines indicate real objects at different depths respectively.

AR technology indeed overlays digital content onto the real world to enhance the user’s perceptions of and interactions with the environment. We performed additional experiments incorporating augmented reality, utilizing a combiner between a waveguide and a camera to concurrently capture both real objects and virtual 3D contents. Figure 9 shows the experimental results of the AR application. In this experiment, we employed the depth-map CGH to simultaneously reconstruct holographic images at multiple depths, as indicated in Figure 9a. Among the virtual contents, an apple was reconstructed at a distance of 150 mm from the SLM, while a grape was reconstructed at a distance of 300 mm from the SLM. Two types of real objects were prepared, a key ring and cup, each located at the same depth as the virtual contents. Figure 9b shows a photo captured when the camera was focused on a near object, i.e., a keyring, and Figure 9c shows a photo captured when the camera was focused on a far object, i.e., a cup. Pictures were captured at a position shifted by −2.3 mm from the center within the expanded eye-box. As shown in Figure 9b, when focusing on a position 150 mm away from the SLM plane, one of the real objects, a key ring, and a virtual image, an apple, are in focus, and the rest appear blurred. In Figure 9c, when focusing on a position 300 mm away from the SLM plane, another real object, a cup, and the remaining virtual object, a grape, are in focus and the rest are out of focus. The results of this experiment indicate that the AR function works effectively by solving visual perception problems caused by depth mismatch between real scenes and virtual content.

**Figure 10:**
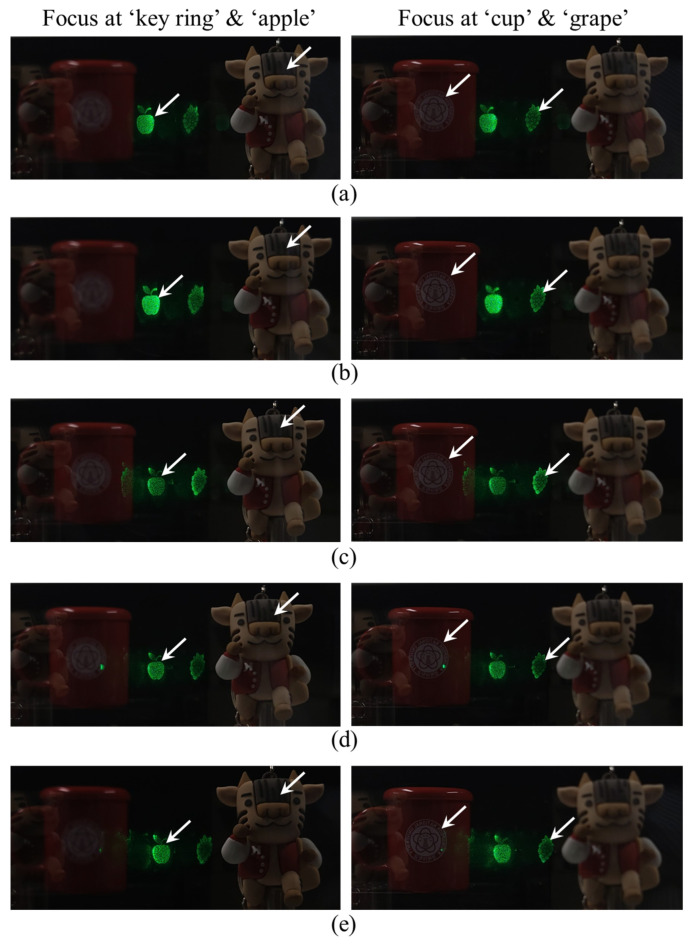
Experimental results of AR application at different pupil positions: (**a**) α=−4.59 mm, (**b**) α=−2.3 mm, (**c**) α=0 mm, (**d**) α=+2.3 mm, and (**e**) α=+4.59 mm. Note that the arrows indicate that the virtual content or real object the camera is focused on.

Figure 10 depicts the experimental results of the AR application at different pupil positions within the expanded eye-box. The position of the shifted eye is the same as that set in Figure 8. In the left photos in Figure 10, the camera was focused on a position 150 mm away from the SLM plane, as shown in Figure 9b. Similarly, in the right photos in Figure 10, the camera was focused on a position 300 mm away from the SLM plane, as shown in Figure 9c. Accommodation-capable virtual images are observed as the eye moves within the expanded eye-box, as shown in Figure 10a–e. These experimental results show that the AR function works effectively by solving the visual perception problem caused by the depth mismatch between the real scene and virtual content within the expanded eye-box.

## 5. Discussion

The proposed lensless holographic waveguide system has no optical components other than a thin waveguide, avoids aberrations caused by optical lenses such as magnifiers, and boasts a compact form factor. An algorithm devised based on an optical equivalent modeling strategy that converts a waveguide into multiple virtual SLMs solves the VAC problem by providing accommodation-capable holographic images with maximum FOV in a waveguide-based system. In addition, an enlarged eye-box is implemented by defining multiple eye reference points and updating the CGH for the moved eye position within the extended eye-box. Most recently, a waveguide-based holographic display with enlarged eye-box that combines the advantages of waveguide display and holography technology was announced [35]. The algorithm described in Ref. [35] involves a complex computational process to model and train coherent optical interactions and propagation within the waveguide to successfully control the output wavefront. Since the waveguide, a key component, uses a grating structure as an input/output coupler, complex modeling is required to compensate for chromatic dispersion and aberration. On the other hand, our study performs optically equivalent modeling using transflective waveguides and boasts a relatively simple computational process via cascade propagation. Therefore, using pupil tracking technology is advantageous in terms of the time it takes to update the CGH of a moved eye position.

In our previous paper, the FOV is extended and the eye-box is proportionally reduced. Therefore, it suffers from a very narrow eye-box since the compensated CGH is not updated according to the position of observer. Since a continuous viewing area must be provided within the eye-box, CGH compensation between viewpoints is essential, and artifacts occurring between viewpoints degrade viewing quality. Additionally, without compensation, only the entire image from a fixed central viewpoint is provided, and adjacent viewpoints introduce brightness and scale errors into the observed image. In this paper, the proposed system generates an accommodation-capable image with a maximum FOV of 7.0 degrees at the optimal observation distance of 112 mm and features an expanded eye-box of 9.18 mm. The size of the expanded eye-box is still not sufficient due to the limited number of virtual SLM replicas. However, this research has great significance in that an expanded eye-box of sufficient size required by the system can be implemented by increasing the number of virtual SLM replicas that contribute to eye reference points according to Equation (4).

It is important to realize that in a lensless holographic system, the maximum FOV is constrained by the pixel pitch of the physical device known as the SLM. While the proposed system successfully achieves a horizontal maximum FOV of 7.0 degrees, there remains significant potential for enhancing the maximum FOV. Efforts are underway both in academia and industry to develop and manufacture SLMs with sub-micrometer pixel pitch. This advancement will provide a future solution to enhancing the maximum FOV in the proposed system. In the long term, accommodation-capable holographic displays based on a waveguide has the potential to synergize with future technological advancements due to their scalability in both FOV and eye-box.

## 6. Conclusions

In this paper, we propose a lensless holographic system with expanded eye-box based on a waveguide consisting of a laser light source, a single SLM, and a waveguide. The proposed system not only generates accommodation-capable images with maximum FOV at the optimal viewing distance, but also expands the eye-box. The optical equivalent modeling strategy of converting the waveguide into multiple virtual SLMs has become an opportunity to break through the limitation of traditional waveguide systems based on the pupil replication process. The virtual SLM replicas generated by this modeling method offer distinct advantages because they define multiple eye reference points at optimal viewing distances. With the help of eye tracking, they contribute to eye-box expansion. Eye-box duplication through the multiplexing method results in visual distortion, either by cutting off part of the image or overlapping repeated images when the eye is positioned between adjacent viewpoints. In contrast, our method effectively expands the eye-box by providing continuous viewpoints within the entire range from the first to the last eye reference point. As a result, the area allowed for eye movement while observing the reconstructed images is sufficiently wide.

In this model, we devised a formalized CGH calculation algorithm based on bold assumption and two constraints and successfully performed numerical observation simulation to observe it. Also, experimental validation of the proposed method was conducted on an optic table. Optical experimental results successfully displayed holographic images with finite depth within an extended eye-box at the optimal observation distance. The maximum horizontal FOV and extended eye-box size were found to be 7.0 degrees and 9.18 mm, respectively, for a total of four virtual SLM replicas at an optimal observation distance of 112 mm. These optical experimental results are consistent with the numerical simulation and support the performance of the proposed algorithm. The results of additional optical experiments successfully reconstructed accommodation-capable virtual contents within the expanded eye-box, fundamentally solving the problem of depth mismatch with the real environment.

## Figures and Tables

**Table 1:** System parameters.

Parameter	Symbol	Value
Wavelength of light source	λ	520 nm
Width of SLM	$W_{SLM}$	4.59 mm (H) × 8.16 mm (V)
Pixel pitch of SLM	p	4.25 μm
Optimal observation distance	$d_{opt}$	112 mm
The number of replicated virtual SLMs	N	4
Distance from the SLM plane to the reconstructed 3D image	$d_{obj}$	200 mm
Maximum FOV	$\Phi_{FOV}$	7.0 degrees
The optical path length difference among virtual SLMs	*l*	7.55 mm
Expanded eye-box size	$W_{eye}$	9.18 mm
Distance between pupil plane and retina plane	$d_{eye}$	25 mm
Radius of pupil	ρ	2 mm

## Data Availability

The data used to support the findings of this study are available from the corresponding author upon request.

## Appendix A. Computer-Generated Hologram Calculation Algorithm

Based on the optical equivalent modeling, the proposed algorithm introduces CdFr and ICdFr within a simplified optical structure that consists of the SLM and the eye [36,37]. CdFr functions by propagating and transforming the modulated wavefront by the SLM in a stepwise manner until it ultimately reaches the retina. CdFr is applied to observation simulation and is important for predicting and analyzing how the CGH will appear to the eye. ICdFr is the opposite process of CdFr and is useful for calculating CGH by tracing the path of light backward from the retina to the SLM.

In the proposed model, the SLM plane $\left(x,y;z_{0}\right)$, pupil plane $\left(x_{p},y_{p};d_{opt}\right)$, and retina plane $\left(x_{r},y_{r};d_{opt}+d_{eye}\right)$ are precisely defined and strategically placed within a coordinated system as shown in Figure 4. We set the SLM plane is placed at $z=z_{0}=0$ plane in the global coordinates. The depth of a reconstructed 3D image in an arbitrary space is determined by the focal length of the eye lens $f_{eye}$, which is expressed as

(A1) $$f_{eye}=\frac{d_{eye}\left(d_{opt}+d_{obj}\right)}{d_{eye}+\left(d_{opt}+d_{obj}\right)},$$

where $d_{eye}$ is the distance between the pupil and retina plane, and $d_{obj}$ denotes the distance from the SLM plane to the reconstructed 3D image. To simplify visual representation, the depth of the holographic image is marked as a constant in Figure 4. However, since the algorithm considers that the depth of the reconstructed 3D image is a variable, it is capable of generating 3D images at multiple depths over the entire range. To expand the eye-box while maintaining maximum FOV at the optimal viewing distance, we calculate the CGH based on the position of the eye shifted by α within the expanded eye-box calculated in Equation (4). When the center of pupil is located on the optical axis, the wavefield of the target image at the retinal plane is expressed as $R\left(x_{r},y_{r};d_{opt}+d_{eye}\right)$. The mathematical relationship between the wavefield at the SLM plane, $S\left(x,y;z_{0}\right)$, and the wavefield at the retina plane of the eye laterally shifted by α, $R\left(x_{r}-\beta ,y_{r};d_{opt}+d_{eye}\right)$, is described as

(A2) $$S\left(x,y;z_{0}\right)=ICdFr\left\{R\left(x_{r}-\beta ,y_{r};d_{opt}+d_{eye}\right);d_{opt},d_{eye},f_{eye}\right\}.$$

Here, $\beta =\left(d_{eye}/d_{opt}\right)\alpha$. For a detailed description of CdFr and ICdFr, please refer to [36] and [37].

Next, we define the relationship between the SLM plane and the retina plane in a lensless holographic waveguide system based on our bold assumption. However, for the actual implementation algorithm, two crucial constraints associated with the pupil replication process should be considered. To compensate for the first constraint, where the optical path from the eye is accumulated each time the virtual SLM is replicated, the wavefield at the SLM plane calculated in Equation (A2) is transferred to the nth virtual SLM. This transfer is achieved via the angular spectrum method (ASM) and ensures that the wavefield on each virtual SLM is acquired correctly. The ASM is defined as follows:

(A3) $$ASM\left\{u\left(x,y;z\right),d\right\}=F^{-1}\left\{F\left\{u\left(x,y;z\right)\right\}exp\left(j2\pi d\sqrt{1/\lambda^{2}-1/{f_{x}}^{2}-1/{f_{y}}^{2}}\right)\right\},$$

where $F\left\{\cdot \right\}$ and $F^{-1}\left\{\cdot \right\}$ denote the Fourier transform and inverse Fourier transform, respectively. Term, $u\left(x,y;z\right)$ is the wavefield at an arbitrary plane parallel to the $\left(x,y;z_{0}\right)$ plane, While $f_{x}$ and $f_{y}$ are spatial frequencies. Therefore, the wavefield on the nth virtual SLM generated in the x-axis, $V_{n}\left(x,y;z_{n}\right)$, is calculated as

(A4) $$V_{n}\left(x,y;z_{n}\right)=ASM\left\{S\left(x,y;z_{0}\right)rect\left[\frac{x+\left(N/2-n\right)W_{SLM}}{W_{SLM}}\right],z_{n}\right\},$$

where $rect\left\{\cdot \right\}$ denotes the rectangular function. This function is used to crop the wavefield at the SLM plane, isolating the region corresponding to the nth virtual SLM with the same number of sampling points as the real SLM. In Equation (A4), when n=0, the wavefield on the real SLM is derived, in which the wavefield $S_{0}\left(x,y;z_{0}\right)$ is expressed as

(A5) $$S_{0}\left(x,y;z_{0}\right)=S\left(x,y;z_{0}\right)rect\left[\frac{x+\left(N/2\right)W_{SLM}}{W_{SLM}}\right].$$

Another constraint is that since virtual SLM replicas are not physical devices, they cannot modulate the wavefield on the entire SLM plane calculated by Equation (A2). To overcome this constraint, a complex wavefield that can be modulated by the real SLM is calculated to achieve the desired effect, similar to it being modulated by each virtual SLM. In our algorithm, the CGH, which is ultimately encoded on the real SLM, $G_{SLM}\left(x,y\right)$, is calculated as follows:

(A6) $$G_{SLM}\left(x,y\right)=S_{0}\left(x,y\right)+\sum_{n=1}^{N}V_{n}\left(x+nW_{SLM},y\right).$$
